# Modulation of Cytokines and Extracellular Matrix Proteins Expression by *Leishmania amazonensis* in Susceptible and Resistant Mice

**DOI:** 10.3389/fmicb.2020.01986

**Published:** 2020-08-31

**Authors:** Flávia de Oliveira Cardoso, Tânia Zaverucha-do-Valle, Fernando Almeida-Souza, Ana Lúcia Abreu-Silva, Kátia da Silva Calabrese

**Affiliations:** ^1^Laboratório de Imunomodulação e Protozoologia, Instituto Oswaldo Cruz, Fundação Oswaldo Cruz, Rio de Janeiro, Brazil; ^2^Laboratório de Anatomopatologia, Departamento de Patologia, Universidade Estadual do Maranhão, São Luís, Brazil

**Keywords:** leishmaniasis, mice, extracellular matrix, immunopathology, cytokines, quantitative PCR, real-time quantitative PCR

## Abstract

Leishmaniases are a complex of diseases with a broad spectrum of clinical forms, which depend on the parasite species, immunological status, and genetic background of the host. In the *Leishmania major* model, susceptibility is associated with the Th2 pattern of cytokines production, while resistance is associated with Th1 response. However, the same dichotomy does not occur in *L. amazonensis*-infected mice. Cytokines are key players in these diseases progression, while the extracellular matrix (ECM) components participate in the process of parasite invasion as well as lesion healing. In this article, we analyzed the influence of host genetics on the expression of cytokines, inducible nitric oxide synthase (iNOS), and ECM proteins, as well as the parasite load in mice with different genetic backgrounds infected by *L. amazonensis*. C57BL/10 and C3H/He mice were subcutaneously infected with 10^6^*L. amazonensis* promastigotes. Lesion kinetics, parasite load, cytokines, iNOS, and ECM proteins expression were measured by quantitative PCR (qPCR) in the footpad, draining lymph nodes, liver, and spleen at early (24 h and 30 days) and late phase (120 and 180 days) of infection. Analysis of lesion kinetics showed that C57BL/10 mice developed ulcerative lesions at the inoculation site after *L. amazonensis* infection, while C3H/He showed slight swelling in the footpad 180 days after infection. C57BL/10 showed progressive enhancement of parasite load in all analyzed organs, while C3H/He mice showed extremely low parasite loads. Susceptible C57BL/10 mice showed high levels of TGF-β mRNA in the footpad early in infection and high levels of proinflammatory cytokines mRNA (IL-12, TNF-α, and IFN-γ) and iNOS in the late phase of the infection. There is an association between increased expression of fibronectin, laminin, collagen III and IV, and TGF-β. On the other hand, resistant C3H/He mice presented a lower repertory of cytokines mRNA expression when compared with susceptible C57BL/10 mice, basically producing TNF-α, collagen IV, and laminin early in infection. The findings of our study indicate that *L. amazonensis* infection induces different cytokine expression in resistant and susceptible mice but not like the *L. major* model. An organ-compartmentalized cytokine response was observed in our model. Host genetics determine this response, which modulates ECM proteins expression.

## Introduction

Leishmaniases are a complex of diseases caused by *Leishmania* spp. It is estimated that between 600,000 and 1 million new cases of cutaneous leishmaniasis (CL) and 50,000 and 90,000 new cases of visceral leishmaniasis (VL) occur worldwide annually (World Health Organization, 2020). *Leishmania amazonensis* is commonly related to the cutaneous manifestations of the disease, although a broad spectrum of clinical manifestations can be found in humans (Barral et al., 1991; Almeida et al., 1996) and mice (Calabrese and Gonçalves da Costa, 1992; Cupolilo et al., 2003). The visceralization of *L. amazonensis* to several organs such as brain (Cupolilo et al., 2003), liver, spleen (Almeida et al., 1996; Abreu-Silva et al., 2004a), and bone marrow (Barral et al., 1986, 1991) has been observed. Reduced cellular immunity, with dysfunction of macrophages and/or T cells is responsible for disease progress (Kong et al., 2017). Development of different clinical manifestations, as well as disease severity, has been attributed, not only to the parasite species, but also to the host genetics and immune responses (Afonso and Scott, 1993; Von Stebut and Udey, 2004; Rosas et al., 2005; Alexander and Brombacher, 2012; Velasquez et al., 2016; Muxel et al., 2018). In comparison to *Leishmania major*, parasites belonging to the *Leishmania mexicana* complex show profound differences in the virulence factors, which affect the immune mechanisms that mediate the susceptibility/resistance to infection (McMahon-Pratt and Alexander, 2004). Transcriptome analysis has shown that different mouse strains present different transcriptional signatures, which might influence the course of *Leishmania* spp. infection. BALB/c and C57BL/6 non-infected macrophages present over 500 genes differentially expressed, which probably influences host response in the event of an infection (Aoki et al., 2019). Similarly, Probst et al. (2012) showed significant differences between C57BL/6 and CBA macrophages baseline gene expression profiles, suggesting that the higher capacity to control *L. amazonensis* infection by C57BL/6 macrophages is due to the baseline transcriptional signature of these cells.

In the 1980s, immunological studies with *L. major* established the Th1/Th2 paradigm of resistance/susceptibility to CL in inbred mice (Heinzel et al., 1989). While BALB/c mice develop a Th2 response, producing IL-4, IL-10, and IL-13, that leads to disease progression, C57BL/6 mice spontaneously heal lesions due to the development of a Th1 response, with the production of IFN-γ (Bogdan and Röllinghoff, 1998; Sacks and Noben-Trauth, 2002; Von Stebut et al., 2003; Von Stebut and Udey, 2004; Alexander and Brombacher, 2012). Balance between Th1 and Th2 responses is primordial for a good prognosis of the disease. Resolution is related to macrophage polarization to the proinflammatory M1 phenotype, characterized by a Th1 response with production of IFN-γ, TNF-α, and granulocyte macrophage colony stimulating factor (GM-CSF). M1 macrophages have increased nitric oxide synthase 2 (NOS2) expression and nitric oxide (NO) levels, which leads to parasite control. On the other hand, disease progression is related to macrophages polarization to an anti-inflammatory M2 phenotype with Th2 cell-mediated production of IL-4, IL-13, IL-10, TGF-β, and macrophage colony-stimulating factor (M-CSF), which increase arginase 1 activity and polyamine production, resulting in parasite replication (Mantovani et al., 2004; Gordon and Taylor, 2005; Lawrence and Natoli, 2011; Wang et al., 2014; Muxel et al., 2018; Aoki et al., 2019;). It is, however, known that cytokines are not exclusive from one or other profile. Early IL-4 release, for instance, is actually important for the establishment of a Th1 response in C57BL/6 mice (Alexander and Brombacher, 2012).

Although it is accepted that protective immunity against *Leishmania* spp. is dependent on a Th1 response, when different inbred mouse strains are infected with New World *Leishmania* species, such as *L. amazonensis*, the Th1/Th2 dichotomy does not occur and the immunological response varies according to the host genetic background (Ji et al., 2002, 2003; Probst et al., 2012). Parasites from the *L. mexicana* complex have different ways of subverting the development of this protective response. Vanloubbeeck et al. (2004) demonstrated that induction of a Th1 phenotype by immunotherapy with dendritic cells in *L. amazonensis*-infected mice does not promote cure, revealing that increase of Th1 cytokines alone does not lead to resistance against this *Leishmania* complex. Nevertheless, it is possible that neutralization of the Th1 response can interfere with protection during *L. amazonensis* infection. *In vitro* and *in vivo* studies showed that *L. amazonensis* promotes the impairment of multiple immune functions at early stages of infection, significantly delaying and depressing the expression of inflammatory cytokines and chemokines (Ji et al., 2003). *In vitro* studies with *L. amazonensis*-infected macrophages have shown that inducible nitric oxide synthase (iNOS) inhibition increases parasite load inside the cells, however, it does not abrograte C3H/He cells resistance to infection (Santos-Pereira et al., 2019).

Immune responses in CL have a critical role during the wound healing process. This process is influenced by the cytokine expression profile (Th1 or Th2) induced by parasite/host interactions. The induction of fibrogenesis has been strongly linked to a Th2 response (with production of IL-4, IL-5, IL-10, and IL-13), while the predominance of IFN-γ and IL-12-producing Th1 cells has been shown to almost completely attenuate the formation of tissue fibrosis (Doucet et al., 1998; Wynn, 2004; Gauglitz, 2013). The main regulatory cytokines involved in this process are IL-10 and TGF-β. At the same time that these cytokines increase the susceptibility to CL, suppressing the immune responses against *Leishmania* parasite (Maspi et al., 2016), they exert a critical role in the acceleration of the wound healing process. IL-10 and TGF-β stimulates the granulation tissue formation, angiogenesis, and extracellular matrix (ECM) synthesis (Gauglitz, 2013; Abdoli et al., 2017).

The ECM is an elaborate meshwork of non-cellular components, basically composed of proteins (e.g., collagens, laminins, fibronectin, and elastin) and proteoglycans (e.g., perlecan and aggrecan). Although it is present within all tissues and organs, its precise composition varies from tissue to tissue (Daley et al., 2008; Frantz et al., 2010; Lu et al., 2011). It provides not only tissue and organ structure but also regulates many aspects of cell behavior, such as cell proliferation and growth, survival, change in cell shape, migration, differentiation, and wound healing (Daley et al., 2008; Hynes, 2009; Lu et al., 2011). The ability of some pathogens to invade host tissues depends on its ability to interact with and overcome a variety of obstacles, including the ECM and basement membrane (BM) proteins, to establish infection within macrophage phagolysosomes (Chang and McGwire, 2002; McGwire et al., 2003). The presence of adhesion molecules on the parasite surface participates in the pathogenesis of leishmaniasis and may explain the affinity of certain species to the skin, where the ECM components are abundant. Parasites of the *L. mexicana* complex present adhesion molecules on their surface, which interact with the ECM during the early stages of infection (Lira et al., 1997; Chang and McGwire, 2002; McGwire et al., 2003; Kulkarni et al., 2008). Promastigotes preferentially bind to type I collagen when they are inoculated into the skin, and this interaction is specific and saturable (Lira et al., 1997). The gp63, a surface protein of *Leishmania*, enhances the *ex vivo* ability of this parasite to migrate through the ECM, degrading components of this matrix, contributing significantly to the access of the parasite at the lymphatic system and blood circulation and facilitates the activation and migration of macrophages (McGwire et al., 2003). In a previous study using mice with different patterns of susceptibility to *L. amazonensis*, our group observed changes in the ECM at the infection site and draining lymph nodes, confirming the involvement of ECM in the host cell-parasite relationship (Abreu-Silva et al., 2004b). These results suggested that amastigotes might be directly involved in the degradation of ECM components as suggested by McGwire et al. (2003).

Therefore, the study of leishmaniasis cellular and humoral immunity, the mechanisms responsible for parasite dissemination in the murine model, as well as the involvement and influence of the ECM in parasite-host relationship are important to evaluate the mechanisms involved in resistance and susceptibility to different species of *Leishmania*. For that reason, the aim of this work was to study the differential modulation of cytokines, iNOS, and ECM components expression in *L. amazonensis*-infected mice from resistant and susceptible genetic backgrounds and their influence on progression or clinical cure of the disease. The study provides a better understanding of the factors responsible for the different clinical forms of the disease and its broad spectrum of immune responses.

## Materials and Methods

### Animals

Four to six weeks old female C57BL/10 (192 animals) and C3H/He mice (192 animals) were obtained from the Instituto de Ciência e Tecnologia em Biomodelos (ICTB/FIOCRUZ) and maintained under pathogen-free conditions, at a controlled temperature and with food and water *ad libitum*. These strains were elected on the basis of their resistance or susceptibility pattern previously determined in our laboratory (Cardoso et al., 2010).

### Parasites

*L. amazonensis* (MHOM/BR/2000/MS501) strain was isolated from the bone marrow aspirate of a patient that developed visceral leishmaniasis in Mato Grosso do Sul state, Brazil, and was maintained by serial passages in BALB/c mice in our laboratory. Periodically, amastigote forms were isolated from mouse footpads and cultivated in LIBHIT medium (Gonçalves da Costa and Lagrande, 1981) to obtain promastigotes for subsequent mouse infection.

### Experimental Design

Mice were divided into four groups with 32 animals each: G1 – infected C57BL/10 mice, G2 – control C57BL/10 mice, G3 – infected C3H/He mice, and G4 – control C3H/He mice. Animals of G1 and G3 were subcutaneously infected by injecting 10^6^ *L. amazonensis* promastigotes in the right hind footpad (RHF) and animals of G2 and G4 were subcutaneously injected with 50 μl of PBS also in the RHF. Infective-stage metacyclic promastigotes were obtained from stationary phase cultures (6 days old) and quantified in a Neubauer hemocytometer prior to infection (Hoff, 1974) using Erythrosine B stain, as described elsewhere (Hodgkinson et al., 1980). Eight animals from each group were euthanized prior to the removal of footpad lesions, draining lymph nodes, spleen, and liver. Organs were obtained at four moments after infection: 24 h and 30 days (early phase), and 120 and 180 days (late phase). Immediately after euthanasia, fragments were removed (approximately 100 mg), immediately frozen, and stored at −70°C. Three independent experiments were performed.

### Lesion Kinetics

Lesion size of the six animals of each group was measured 1, 30, 120, and 180 days after *L. amazonensis* infection, using a Schnelltaster dial gauge caliper (Kröplin GRBH). The footpad swelling was expressed as the difference in thicknesses in millimeters between the inoculated footpad and normal contralateral footpad.

### qPCR Primer Design

All primers were designed to be used in a SYBR green quantitative PCR (qPCR) assay for detection and quantification of *Leishmania* DNA and cytokines, iNOS and ECM proteins mRNA expression. Primers targeting *Leishmania* kinetoplast minicircle 3 (kDNA3) and *Actb – actin, beta* (β-actin) mouse gene were synthesized as previously reported (Giulietti et al., 2001; Weirather et al., 2011). Primers targeting the mouse genes, *Il4 – interleukin 4, transcript variant 1* (IL-4); *Il10 – interleukin 10* (IL-10); *Il12a – interleukin 12a, transcript variant 1* (IL-12); *Tnf – tumor necrosis factor, transcript variant 1* (TNF-α); *Ifng – interferon gamma* (IFN-γ); *Tgfb1 – transforming growth factor, beta 1* (TGF-β); *Nos2 – nitric oxide synthase 2, inducible, transcript variant 1* (iNOS); *Lama5 – laminin, alpha 5* (LM); *Fn1 – fibronectin 1, transcript variant 1* (FN); *Col1a1 – collagen, type I, alpha 1* (collagen I); *Col3a1 – collagen, type III, alpha 1* (collagen III); *Col4a2 – collagen, type IV, alpha 2* (collagen IV); and *Rplp0 – ribosomal protein, large, P0* (*Rplp0*), were designed using the Primer Express software version 3.0 (Applied Biosystems, 2004; Table 1). All primers were manufactured by Gene Link (Hawthorne, NY). The relative quantification and calibration of the mRNA levels was performed using the mouse gene *Rplp0* as the endogenous control. For parasite quantification, the *Actb* reference gene was used as a positive control to monitor DNA integrity, the presence of potential inhibitors of PCR or variation in DNA yield.

**Table 1:** Primers used for real time PCR.

Target	Primer sequence		Sequence source
	Forward	Reverse	
kDNA3	GGGTAGGGGCGTTCTGC	CCCGGCCTATTTTACACCAACC	M94088.1
β-actin	AGAGGGAAATCGTGCGTGAC	CAATAGTGATGACCTGGCCGT	X03672.1
IL-4	TTGAACGAGGTCACAGGAGAAG	AGGACGTTTGGCACATCCA	NM_021283.2
IL-10	GATGCCCCAGGCAGAGAA	CACCCAGGGAATTCAAATGC	NM_010548.2
IL-12	ACAGGGTGATGGGCTATCTGA	TGTGGCAGAGGGCCTTGA	NM_001159424.2
TNF-α	CACAAGATGCTGGGACAGTGA	TCCTTGATGGTGGTGCATGA	NM_013693.3
IFN-γ	TTGGCTTTGCAGCTCTTCCT	TGACTGTGCCGTGGCAGTA	NM_008337.4
TGF-β	GCAGTGGCTGAACCAAGGA	AGCAGTGAGCGCTGAATCG	NM_011577.2
iNOS	GGATCTTCCCAGGCAACCA	CAATCCACAACTCGCTCCAA	NM_010927.4
Laminin	GCAGGACGACGACGTCATCT	AAGTCTCGAAGTAACGGTGAGTAGGA	NM_001081171.2
Fibronectin	GTGTAGCACAACTTCCAATTACGAA	GGAATTTCCGCCTCGAGTCT	NM_010233.2
Collagen I	CTTCACCTACAGCACCCTTGTG	TGACTGTCTTGCCCCAAGTTC	NM_007742.3
Collagen III	AAGGCGAATTCAAGGCTGAA	TGTGTTTAGTACAGCCATCCTCTAGAA	NM_009930.2
Collagen IV	ACGGGCCAACGCTTCTTC	CATGATCCCAGTCTTTGAGCTCTA	NM_009932.4
Rplp0	GCCAGCTCAGAACACTGGTCTA	ATGCCCAAAGCCTGGAAGA	NM_007475.5

### DNA and mRNA Extraction

For DNA extraction, fragments from the lesion site (footpad), draining lymph node, spleen, and liver were digested in 500 μl of lysis buffer (50 mM Tris, 10 mM NaCL, 5 mM EDTA, 0,5% SDS) containing proteinase K (20 mg/ml). DNA was extracted following a standard phenol/chloroform protocol (Sambrook and Russell, 2006).

Total RNA from tissues was extracted using the TRIZOL reagent (Invitrogen, Karlsruhe, Germany) following the manufacturer’s instructions. cDNA synthesis was performed with 1 μg of total RNA using iScript cDNA Synthesis kit (Bio-Rad Laboratories, Hercules, CA), according to the manufacturer’s recommendations.

DNA, mRNA, and cDNA concentrations and purity were determined by reading A260 and A280 on a NanoDrop 2000c spectrophotometer (Thermo Fisher Scientific, Wilmington, DE, United States).

### qPCR Assay Conditions

All reactions were conducted using Applied Biosystems 7500 Fast Real-Time PCR Systems.

For the *Leishmania* DNA quantification, the reaction mixtures contained Fast SYBR Green Master Mix 2X (Applied Biosystems), 250 nM of kDNA3 or 100 nM of β-actin primers and 20 ng of DNA template in a final volume of 20 μl. PCR conditions were as follows: hold at 95°C for 20 s, followed by 40 temperature cycles of 95°C for 3 s and 60°C for 30 s. Standard curves were generated from 10-fold serial dilutions of axenic *Leishmania* DNA (100 ng–1 pg).

For ECM proteins, iNOS and cytokines qPCR assay, the reaction mixtures contained 12.5 μl of Power SYBR Green PCR Master Mix (Applied Biosystems) with 5 μl of cDNA (15 ng) for all targets, except IL-4 and collagen IV for which 10 μl were used; 100 nM of each primer was used in each reaction in a final volume of 25 μl. The temperature parameters consisted of a hold at 95°C for 10 min followed by 40 temperature cycles of 95°C for 15 s and 58°C for 1 min.

A melt curve analysis was performed on all reactions. The quality parameters of the standard curves were analyzed with the 7500 software v2.0.6 (Applied Biosystems).

### Statistical Analysis

The data were expressed by mean ± SD and analyzed statistically by two-way ANOVA and Bonferroni’s multiple comparison test. Correlation between parasite load and lesion kinetics was performed by Pearson’s linear correlation coefficient in each strain. The analyses were performed with the software GraphPad Prism 6.01 and differences were considered significant when *p* < 0.05.

## Results

### Parasite Load and Lesion Kinetics

**Figure 1:**
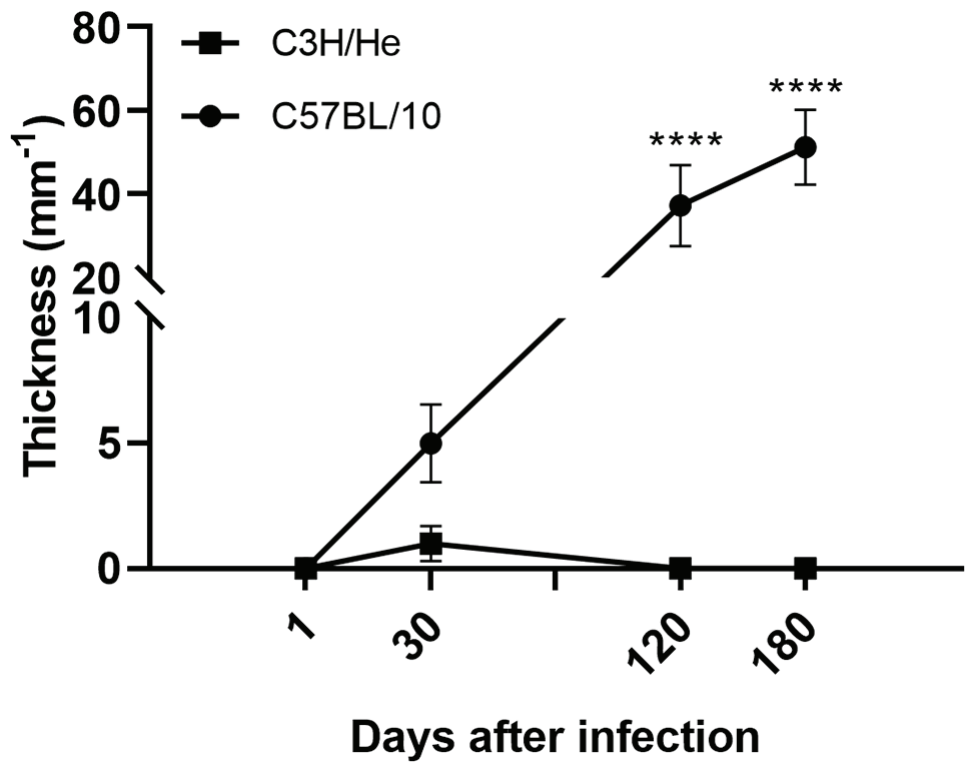
Lesion kinetics. Progression of lesion size in the footpad of C57BL/10 and C3H/He mice subcutaneously infected with 10^6^ *Leishmania amazonensis* promastigote forms in the right hind footpad (RHF). Data represent mean ± SD of two independent experiments with four animals. ^****^p < 0.0001 when compared between groups by two-way ANOVA and Bonferroni’s multiple comparison test.

Primary lesion kinetics showed that C57BL/10 mice developed ulcerative and progressive cutaneous lesions after *L. amazonensis* infection, while C3H/He infected mice presented a discrete footpad swelling, that never developed into lesions even 180 days after infection (Figure 1).

**Figure 2:**
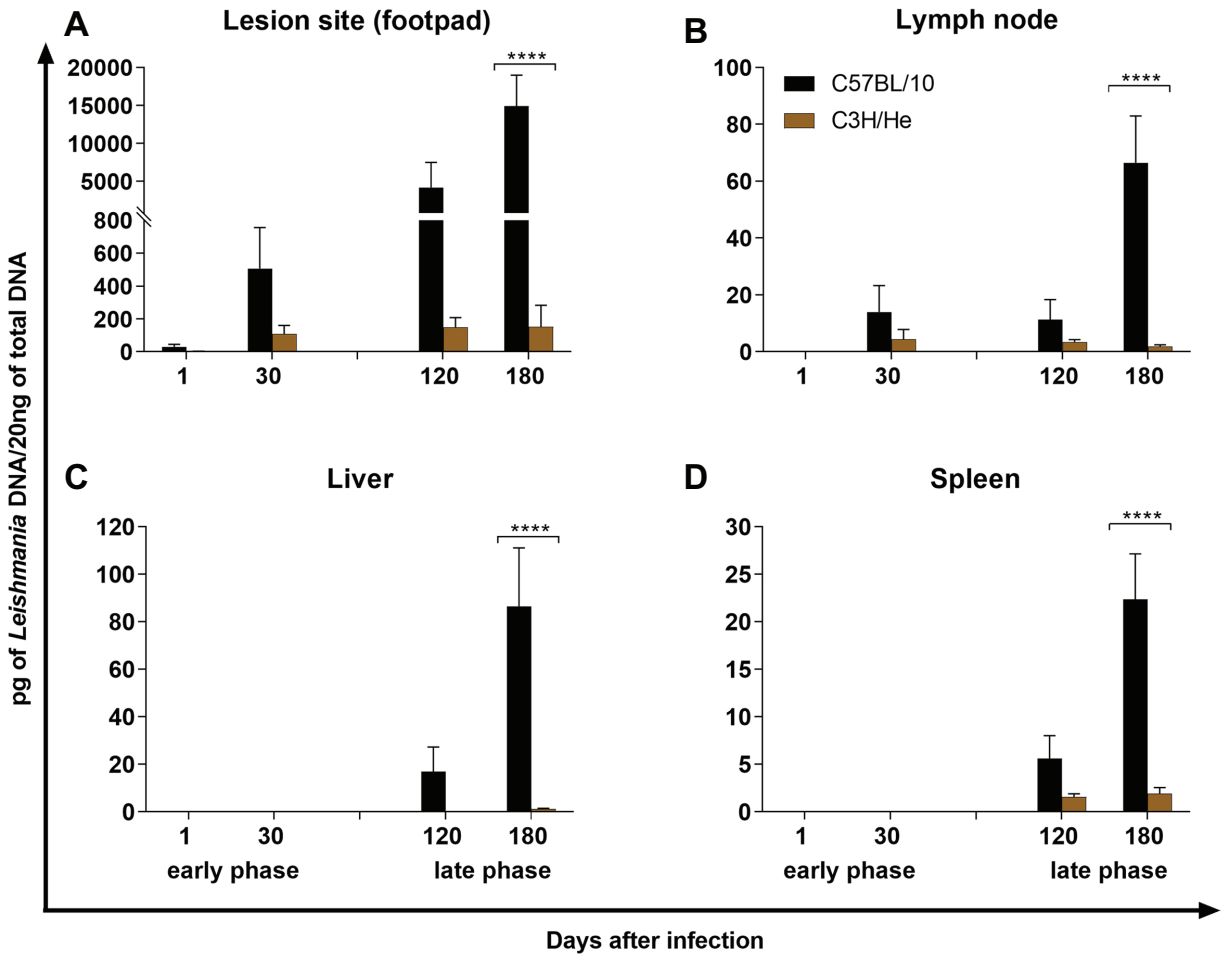
Parasite Load. Quantification of parasites by quantitative PCR (qPCR) in the footpad (lesion site; **A**), draining lymph node **(B)**, liver **(C)**, and spleen **(D)** of C57BL/10 and C3H/He mice subcutaneously infected with 10^6^ *L. amazonensis* promastigote forms in the RHF. Data represent mean ± SD of two independent experiments with four animals assayed in triplicate. ^****^p < 0.0001 when compared with the control group or between groups in brackets by two-way ANOVA and Bonferroni’s multiple comparison test.

qPCR analysis showed a progressive enhancement of parasite load in C57BL/10 in all organs analyzed (Figure 2). Parasite density was higher in the lesion site (footpad), but parasite DNA was present in all organs. C3H/He mice showed extremely low parasite loads in all analyzed tissues. Parasite visceralization to liver and spleen occurred in C57BL/10 mice, at the late phase of infection. These mice showed a growing parasite load in both organs from 120 days after infection, while C3H/He mice showed a low parasite load in spleen at the late phase of infection. A positive correlation between parasite load in the footpad and lesion size was found in C57BL/10 mice (*r* = 0.9519, *p* = 0.0481) but not in C3H/He (*r* = 0.6323, *p* = 1838).

### Cytokines and iNOS Expression Analysis

**Figure 3:**
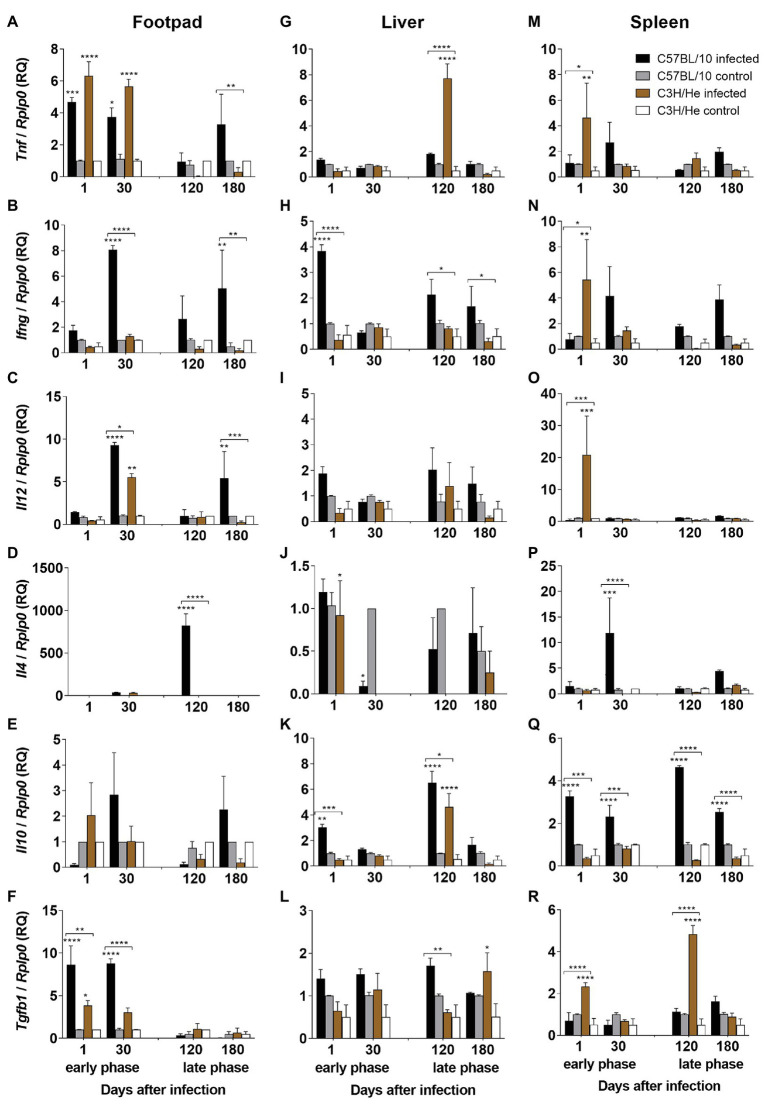
Cytokine expression profile. Cytokine expression by real-time quantitative PCR (RT-qPCR) in the footpad (lesion site), liver, and spleen of C57BL/10 and C3H/He mice subcutaneously infected with 10^6^ *L. amazonensis* promastigote forms in the RHF and normal mice from the same genetic background. Expression of TNF-α **(A,G,M)**, IFN-γ **(B,H,N)**, IL-12 **(C,I,O)**, IL-4 **(D,J,P)**, IL-10 **(E,K,Q)**, and TGF-β **(F,L,R)** was estimated by ΔΔCT method, using *Rplp0* as a reference gene. Data represent mean ± SD of two independent experiments with four animals assayed in triplicate. ^*^p < 0.05, ^**^p < 0.01, ^***^p < 0.001, and ^****^p < 0.0001 when compared with the control group or between groups in brackets by two-way ANOVA and Bonferroni’s multiple comparison test. RQ, relative quantification; *Ifng – interferon gamma*; *Tnf – tumor necrosis factor, transcript variant 1*; *Il12a – interleukin 12a, transcript variant 1*; *Il4 – interleukin 4, transcript variant 1*; *Il10 – interleukin 10*; *Tgfb1 – transforming growth factor, beta 1; Rplp0 – ribosomal protein, large, P0*.

**Figure 4:**
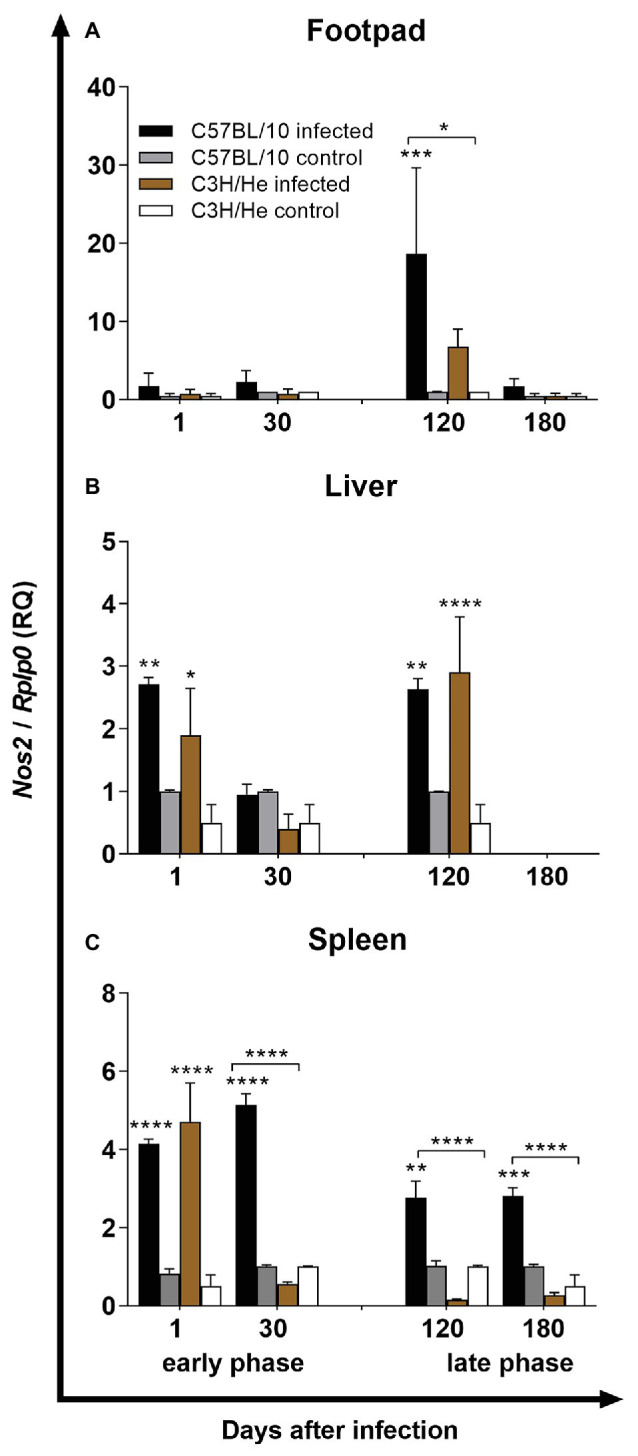
Inducible nitric oxide synthase (iNOS) expression. iNOS expression by RT-qPCR in the footpad (lesion site; **A**), liver **(B)**, and spleen **(C)** of C57BL/10 and C3H/He mice subcutaneously infected with 10^6^ *L. amazonensis* promastigote forms in the RHF and normal mice from the same genetic background. Expression of iNOS was estimated by the ΔΔCT method, using *Rplp0* as a reference gene. Data represent mean ± SD of two independent experiments with four animals assayed in triplicate. ^*^p < 0.05, ^**^p < 0.01, ^***^p < 0.001, and ^****^p < 0.0001 when compared with the control group or between groups in brackets by two-way ANOVA and Bonferroni’s multiple comparison test. RQ, relative quantification; *Nos2 – nitric oxide synthase 2; inducible, transcript variant 1; Rplp0 – ribosomal protein, large, P0*.

Analyses of footpads, in the early phase of infection, showed that *L. amazonensis* infection induced the upregulation of TNF-α and TGF-β expression in both mouse strains, when compared with control groups, 24 h after infection. The expression of TGF-β mRNA-transcripts was significantly higher in C57BL/10 than in C3H/He mice at this time (Figures 3A,F). After 30 days of infection, both infected mouse strains showed an upregulation of TNF-α, IL-12, and TGF-β, but IL-12 and TGF-β upregulation was higher in C57BL/10 mice compared to C3H/He mice (Figures 3A,C,F). IFN-γ mRNA upregulation was observed only in C57BL/10 infected mice (Figure 3B). In the late phase of infection, after 120 days of *L. amazonensis* inoculation, an upregulation of IL-4 and iNOS expression was observed in C57BL/10 when compared with C3H/He mice (Figures 3D, 4A). At 180 days, C57BL/10 mice showed an upregulation of proinflammatory cytokines (TNF-α, IFN-γ, and IL-12) in comparison with C3H mice (Figures 3A–C). No differences in IL-10 mRNA expression was observed in the footpads (Figure 3E). These results indicate a strong Th1 stimulus, with the upregulation of proinflammatory cytokines mRNA-transcripts in C57BL/10 infected mice when compared with C3H/He infected mice in the early and late phases of infection, suggesting an unbalance of immune response, which might contribute to excessive tissue damage in that strain.

The cytokine evaluation in the draining lymph node showed low cytokine expression. On the next day after infection, an upregulation of iNOS and TGF-β could be observed in C57BL/10 mice followed by an increase of those genes and the inflammatory cytokines TNF-α, IFN-γ, and IL-12, 30 days after infection. At that time, expressions of IL-10 and IL-4 could only be detected in C57BL/10. At later times, no modulation was observed in neither mouse strain (Supplementary Table S1). These results showed a mixed immune response in draining lymph nodes of C57BL/10 infected mice in early phase of infection and low cytokine expression in late phase in both mouse strains.

In the liver, on the first day after infection, an upregulation of IFN-γ and IL-10 expressions was observed in susceptible C57BL/10 mice when compared with C3H/He mice (Figures 3H,K, respectively). IL-4 was upregulated in C3H/He infected mice, in comparison with non-infected mice (Figure 3J). Finally, iNOS expression was upregulated in both mouse strains, when compared with control groups, but no significant difference was observed between the infected lineages (Figure 4B). On the thirtieth day after infection, a significant IL-4 mRNA downregulation was observed in C57BL/10 infected mice when compared with the control group (Figure 3J). In the late phase, 120 days after *L. amazonensis* infection, C57BL/10 mice presented an increase of IFN-γ, IL-10, and TGF-β expressions in comparison with C3H/He mice (Figures 3H,K,L). C3H/He mice also showed an increase in IL-10 expression in comparison to the control group, in addition to an upregulation of the TNF-α mRNA transcript, which proved to be significantly higher than in C57BL/10 infected mice (Figures 3G,K, respectively). The iNOS expression increased in both infected mouse strains, only when compared with their controls (Figure 4B). After 180 days, few transcripts were regulated. TNF-α, IL-12, IL-4, IL-10, and iNOS showed basal levels of expression on both infected lineages (Figures 3G,I–K, 4B). C57BL/10 mice showed the same profile of IFN-γ expression observed at 120 days, which were significantly higher than in C3H/He infected mice (Figure 3H), whereas C3H/He infected mice, showed an upregulation of TGF-β expression when compared with the control group (Figure 3L). Altogether, these results showed a higher regulation of mRNA-transcripts in the liver by C57BL/10 infected mice, mainly in the late phase of infection, where parasites can be detected in this organ.

The spleen analysis showed a significant enhancement of proinflammatory cytokines expression – TNF-α, IFN-γ, and IL-12 – and regulatory cytokine – TGF-β – on the first day after infection in C3H/He mice, in comparison with C57BL/10. All these cytokines returned to basal levels at later times, except for TGF-β, which was upregulated again 120 days after infection (Figures 3M–O,R). IL-10 expression in C57BL/10 infected mice was significantly upregulated during the experiment (Figure 3Q), while IL-4 expression was upregulated in this group only at 30 days after infection (Figure 3P). The iNOS expression on both mice strains was upregulated by *L. amazonensis* 24 h after infection, decreasing to basal levels in C3H/He mice at later times but remaining upregulated in C57BL/10 mice (Figure 4C). Altogether, these results showed that *L. amazonensis* infection causes an impairment in the immune response in the spleen of C57BL/10 mice during all infection, with higher upregulation of IL-10, mainly at later times where the parasite load is high. On the other hand, C3H/He infected mice showed an early Th1 proinflammatory response, which might have been important for infection control.

### Extracellular Matrix Component Analysis

**Figure 5:**
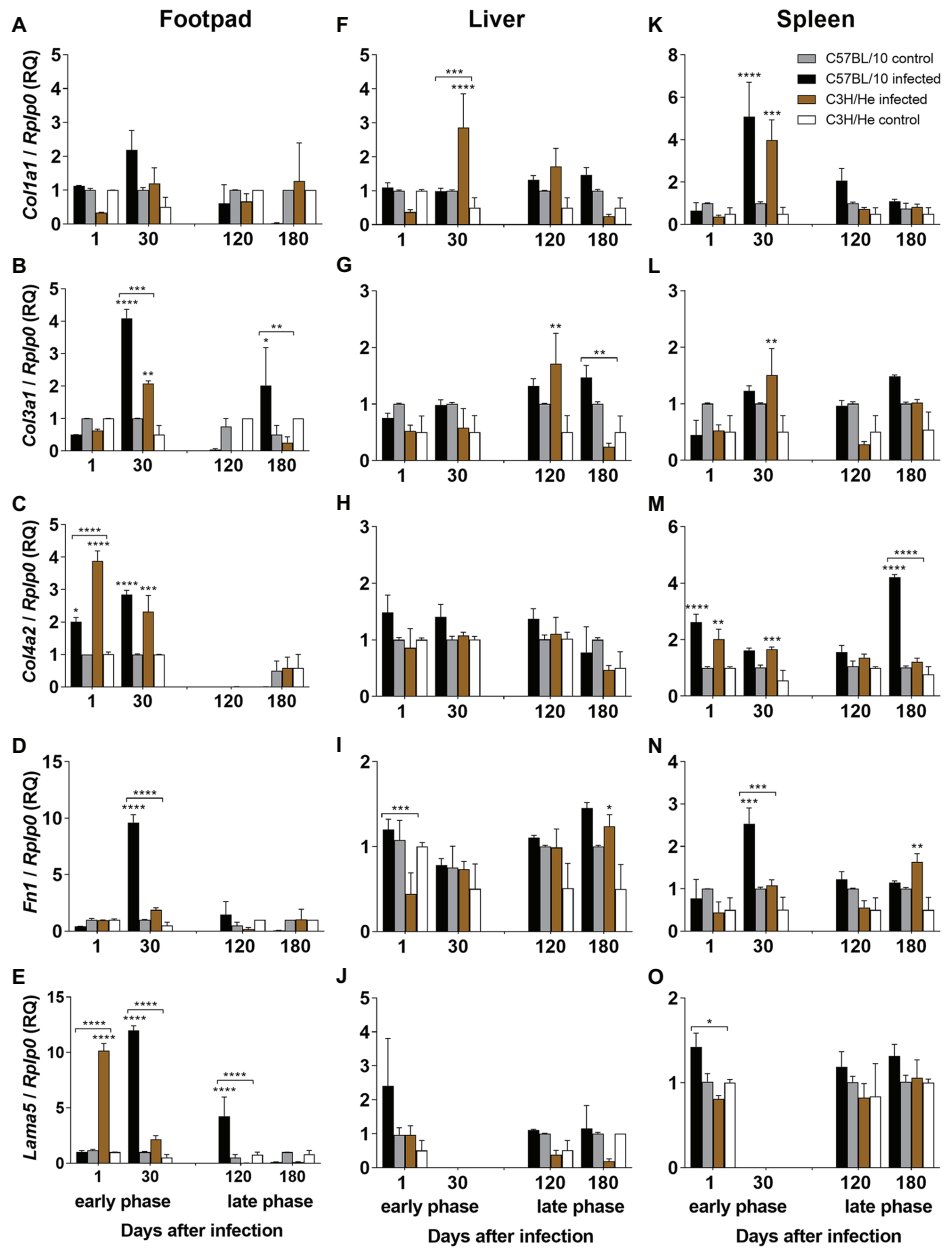
Extracellular matrix (ECM) protein expression. Expression profile of ECM proteins by RT-qPCR from the lesion site, liver, and spleen of C57BL/10 and C3H/He mice subcutaneously infected with 10^6^ *L. amazonensis* promastigote forms in the RHF and normal mice from the same genetic background. Expression of collagens I **(A,F,K)**, III **(B,G,L)**, and IV **(C,H,M)**, fibronectin **(D,I,N)**, and laminin **(E,J,O)** were estimated by the ΔΔCT method, using *Rplp0* as a reference gene. Data represent mean ± SD of two independent experiments with four animals assayed in triplicate. ^*^p < 0.05, ^**^p < 0.01, ^***^p < 0.001, and ^****^p < 0.0001 when compared with the control group or between groups in brackets by two-way ANOVA and Bonferroni’s multiple comparison test. RQ, relative quantification; *Col1a1 – collagen, type I, alpha 1*; *Col3a1 – collagen, type III, alpha 1*; *Col4a2 – collagen, type IV, alpha 2*; *Fn1 – fibronectin 1*; *Lama5 – laminin, alpha 5*, *transcript variant 1; Rplp0 – ribosomal protein, large, P0*.

Footpad real-time quantitative PCR (RT-qPCR) analyses showed, in the early phase of infection, an upregulation of collagen IV mRNA-transcripts after 1 day of *L. amazonensis* infection in both mouse strains, but this upregulation was significantly higher in C3H/He infected mice (Figure 5C), which also showed only a transient increase of LM (Figure 5E). Thirty days after infection, the expression levels of collagen III, IV, FN, and LM were upregulated in C57BL/10 infected mice (Figures 5B–E). At the same time, in C3H/He mice only collagens III and IV were upregulated (Figures 5B,C), decreasing at basal levels in the late phase of infection. In the late phase of infection, only LM (120 days) and collagen III (180 days) were upregulated in C57BL10/10 infected mice (Figures 5B,E, respectively). Collagen I expression maintained basal levels throughout the experiment (Figure 5A). These results showed an upregulation of BM components (Collagen IV and FN) in the early phase of infection, mainly 1 day after *L. amazonensis* inoculation, suggesting a degradation of these components by parasite and consequent tissue invasion. In the late phase of infection, few ECM components were expressed, either by complete wound healing (C3H/He) or by excessive tissue destruction (C57BL/10).

The lymph node evaluation showed low levels of mRNA from ECM proteins at all times after infection in both mouse strains. The exception was a higher expression of collagens I, III, and IV in C57BL/10 mice 30 days after infection (Supplementary Table S1).

In the liver, *L. amazonensis* infection induced a downregulation of fibronectin-mRNA in C3H/He mice when compared with C57BL/10 mice 1 day after infection and a mild upregulation after 180 days in C3H/He infected mice in comparison with the control group (Figure 5I). An increase of collagen I mRNA was observed in *L. amazonensis*-infected C3H/He mice only 30 days after infection (Figure 5F). A hundred and twenty days after infection, there was an upregulation of collagen III in C3H/He mice when compared with the control group and, after 180 days, there was a downregulation of C3H/He mRNA expression in comparison with *L. amazonensis*-infected C57BL/10 mice (Figure 5G). Both mouse strains maintained basal levels of collagen IV and LM expression throughout the experiment (Figures 5H,J). These results showed that, in spite of liver colonization, infection caused only a mild modulation in the expression of ECM components, which suggests that tissue architecture was not intensely affected by the parasite presence.

Spleen analysis showed that one day after infection, collagen IV expression was enhanced in both infected mouse strains but gradually decreased at later times, to increase again 180 days after infection, only in C57BL/10 (Figure 5M). C57BL/10 infected mice also showed an upregulation of LM when compared with C3H/He (Figure 5O). Thirty days after infection, there was an increase of collagen I mRNA expression on both mice strains (Figure 5K) and an upregulation of collagens III and IV expression in C3H/He infected mice when compared with the control group (Figures 5L,M). At the same time, the FN expression in C57BL/10 infected mice was higher when compared with C3H/He (Figure 5N). On the opposite, after 180 days of infection, there was an upregulation of this ECM protein expression in C3H/He when compared with the control group (Figure 5N). These observations suggest that like observed in the footpad, the spleen showed an upregulation of BM components (Collagen IV and LM) in the early phase of infection, 1 day after *L. amazonensis* inoculation, mainly in C57BL/10 infected mice. C3H/He infected mice showed an upregulation of collagens expression at 30 days after infection. In the late phase of infection, despite the parasite detection in this organ, there were few ECM proteins expressions.

## Discussion

Immunological mechanisms of leishmaniasis are complex and the outcome of infection depends on the association of host genetics and *Leishmania* species, which influence the type of immune response promoting healing or disease progression. Promastigote forms of *Leishmania* are regurgitated by the phlebotomine at the moment of blood meal and immediately get in touch with the extracellular microenvironment interacting with ECM constituents (Ghosh et al., 1999). Skin is the largest organ of the body and acts not only as a physical barrier against infection but also as an important immunological organ (Nylén and Eidsmo, 2012; Abdoli et al., 2017). After the skin injury produced by sandfly bites and *Leishmania* deposition, several growth factors and cytokines, including TGF-β, are released to attract immune (e.g., neutrophils, macrophages, and eosinophil) and non-immune cells (e.g., fibroblasts and endothelial cells; Gauglitz, 2013). Neutrophils rapidly infiltrate the wounded skin and take up the parasites (Laskay et al., 2008; Peters et al., 2008; Abdoli et al., 2017). Posteriorly, within 2 days, monocytes/macrophages infiltrate to infection site and act as the main host cells for *Leishmania* amastigotes (Ribeiro-Gomes et al., 2004; van Zandbergen et al., 2004; Peter et al., 2007; Abdoli et al., 2017). During the early phase of infection, no macroscopic alterations occur in the epidermis. The development of ulcerative lesions is followed by a vigorous acute inflammatory response (Modlin et al., 1989) and dermal necrosis, accompanied by a reduction in the parasite number in the dermis (Lima et al., 1994). At this stage, infiltrating T cells produce IL-10, TNF-α, and IFN-γ (Belkaid et al., 2002; López et al., 2012). Depending on the infecting *Leishmania* species and host genetics, lesions progress into erythematous nodules, indurated plaques, scaly plaques, or ulcers (Nylén and Eidsmo, 2012). The relationship between parasite growth, lesion formation, and immunity has been studied by several authors in different mouse strains (Lira et al., 2000; Cardoso et al., 2010; Velasquez et al., 2016; Souza et al., 2018). Velasquez et al. (2016) have shown that *L. amazonensis*-infected C57BL/6 mice developed smaller lesions that progressed to complete healing, while BALB/c and BALB/c nude mice developed progressive lesions, which correlated to the parasite load. In the present study, a positive correlation between parasite load and lesion size was also found in C57BL/10 mice but not in C3H/He. C57BL/10 mice developed ulcerative and progressive cutaneous lesions after *L. amazonensis* infection, while C3H/He infected mice presented a discrete footpad swelling and never developed lesions even at the end of infection.

Oxide nitric is a key player in the control of *Leishmania* parasites. As early as 24 h after infection, iNOS expression is induced by type 1 IFN. Antibody blockage of IFNα/β at that point abolishes iNOS expression, leading to parasite dissemination (Diefenbach et al., 1998). At later times of infection, iNOS expression is induced by IFN-γ in combination with TNF. *In vitro*, macrophages treated with IFN-γ alone or in combination with TNF-α, rapidly upregulate iNOS expression and produce NO (Liew et al., 1991). Overall, NO production by iNOS promotes *Leishmania* control from the first hours after infection, until the resolution of the clinical phase and control of parasite latency at the healed tissue (Olekhnovitch and Bousso, 2015). In the present work however, resistant C3H/He presented lower levels of iNOS than susceptible C57BL/10, suggesting that resistance is not dependent on NO production by iNOS. In fact, iNOS inhibition in C3H/He peritoneal macrophages did not abrogate the resistant phenotype. C3H/He macrophages maintained a heavier parasite load than iNOS-inhibited BALB/c macrophages (Santos-Pereira et al., 2019).

Although Th1 response is required to induce lesion healing in CL caused by *L. major* in humans and mice (Kane and Mosser, 2001; Murray et al., 2002; Trinchieri et al., 2003), excessive proinflammatory cytokines, mainly IFN-γ and TNF-α, can contribute to development of mucosal leishmaniasis (LM) (Follador et al., 2002) and to the tissue damage (Ribeiro-de-Jesus et al., 1998) produced by cellular recruitment, as observed in infections caused by *L. braziliensis* (Vargas-Inchaustegui et al., 2010) and *L. amazonensis* (Carneiro et al., 2015). Studies conducted in mock-treated C57BL/6 *L. amazonensis*-infected mice showed an excessive secretion of pro-inflammatory cytokines in these animals, leading to uncontrolled tissue degradation and perpetuates the non-healing condition (Almeida-Souza et al., 2016). However, the production of regulatory cytokines, mainly IL-10, is related to the control of exacerbated inflammatory response. Antonelli et al. (2004) using peripheral blood mononuclear cell (PBMC) cultures from patients with localized cutaneous leishmaniasis (LCL), stimulated with soluble *Leishmania* antigens demonstrated a strong positive correlation between IL-10 and TNF-α, suggesting an active mechanism of intrinsic self-regulation of the macrophage in these patients. On the other hand, patients with LM showed a decreased expression of IL-10 receptors (Faria et al., 2005), which can lead to a poorly controlled inflammatory response. Such observations corroborate the data obtained in our study in the primary lesion, where the most susceptible mice (C57BL/10) presented, in the late stage of infection, a higher expression of proinflammatory cytokines such as IL-12, IFN-γ, and TNF‐ α. These higher proinflammatory cytokine and the lack of IL-10 expression can contribute to footpad swelling, presence of intense inflammatory infiltrate and severe tissue damage, as observed previously in this strain by our group in the late phase of infection (Cardoso et al., 2010; Souza et al., 2018).

It is known that *L. amazonensis* parasites have unique strategies to delay the induction of host immune responses at early stages, favoring its establishment and disease pathogenesis at later stages of infection (Ji et al., 2003). The parasite is able to subvert the host response at multiple levels including modification of cytokines, chemokines, and their receptor expression, preventing antigen presentation by major histocompatibility complex (MHC) class II molecules and killing mechanisms through reduction of NO production. This impairment of immune response, early in infection, favors parasite establishment and development of chronic disease (Ji et al., 2003; Aoki et al., 2019). Several studies showed that *L. amazonensis* induces TGF-β production early in infection. It is frequently seen in the literature that TGF-β acts suppressing the immune response and macrophage activation (Tsunawaki et al., 1988), increasing the susceptibility to CL (Maspi et al., 2016; Abdoli et al., 2017). Consistent with this view, Barral-Netto et al. (1992) showed an increased production of TGF-β in *L. amazonensis*-infected BALB/c footpads and peritoneal macrophages. In addition, they have also shown an enhancement of lesion development in mice receiving exogenous TGF-β. These results demonstrate that TGF‐ β exerts inhibitory effects on a macrophage’s ability to kill intracellular pathogens. Other studies reinforce this idea, showing that TGF-β blocks IFNγ-induced macrophages activation, reducing their oxidative responses (Ding et al., 1990). Besides, TGF-β is also involved in the attraction of immune cells (e.g., neutrophils, macrophages, epithelial cells, and mast cells) to the site of injury (Gauglitz, 2013; Abdoli et al., 2017), which is often associated with increased pathology (Alexander and Brombacher, 2012). In previous studies (Cardoso et al., 2010), we showed that footpads of C57BL/10 mice presented an intense inflammatory infiltrate composed by vacuolated macrophages full of amastigotes, mast cells, and eosinophils in the initial phase of infection and these mice developed progressive ulcerative lesions. These results added to the present findings, where an enhancement of TGF-β expression in C57BL/10 footpads (early in infection) was observed, sustains the hypothesis that this cytokine contributes to macrophage failure to kill parasites, which in turn leads to chronic inflammatory responses and tissue destruction, favoring disease progression in C57BL/10 infected mice.

The spleen is a main organ involved in the immune response of VL. As early as the first 24 h after infection, dendritic cells containing parasite antigens, but not parasites, secrete IL-12, and prime T-cell, inducing their differentiation on Th1 cells, which, in turn will produce pro-inflammatory cytokines such as TNF-α and IFN-γ, maximizing the capacities of macrophages to produce NO and reactive oxygen species to fight parasites (Rodrigues et al., 2016). In *L. infantum*-infected dogs, parasite load negatively correlates with pro-inflammatory (IFN-γ, IL-12, and IL-6) and anti-inflammatory (IL-10 and TGF-β) cytokine levels on the spleen (Cavalcanti et al., 2015). Similarly, the evolution of the infection process in dogs may be determined correlating the parasite load with the TNF-α and IL-10 level in the spleen (Michelin et al., 2011). In the present study, infected C57BL/10 mice showed high levels of IL-10, which was also associated with parasite presence in this organ. In fact, IL-10 produced by T reg cells have been implicated in the persistence of *L. major* parasites in infected C57BL/6 mice. In the absence of IL-10, however, sterile cure occurs (Alexander and Brombacher, 2012). In addition, a study using B-1CDP cells infected by *L. major* showed that cells from wild type mice showed higher parasite loads than cells from IL-10 KO mice, confirming the importance of IL-10 in preventing parasite killing (Arcanjo et al., 2015). On the other hand, IL-10 production by B-2 lymphocytes, but not by B-1 lymphocytes, have been related with faster lesion progression during *L. amazonensis* infection (Firmino-Cruz et al., 2018, 2020). While no parasite was noted in the C3H/He spleen when limiting dilution assay was employed (Cardoso et al., 2010), the presence of *Leishmania* DNA was revealed in the present study by qPCR. Pathogen-derived nucleic acids could be recognized by host PRRs and the cross-linked of these molecules triggers signaling cascades that culminate in the cytokine production, leading to activation of immunity (Tan et al., 2018). In this way, the presence of *Leishmania* DNA can explain the cytokine profile found in C3H/He mice. On the other hand, C3H/He spleen showed an early response to infection, with high expression of TNF-α, TGF-β, IL-12, IFN-γ, and iNOS, suggesting a commitment of the spleen in the early response against infection. It is known that spleen macrophages demonstrate a remarkable innate capacity to kill *L. donovani* parasites, reducing in 50% the inoculum in the first 24 h after infection (Rodrigues et al., 2016). This rapid spleen response may be related with C3H/He resistance.

The liver is considered an important immune tissue that acts on the front line, ideally positioned to detect pathogens. Dynamic interactions between the numerous immune cell populations in the liver are the key to maintain balance and overall tissue health. Studies on VL suggest that central target organs, such as the liver and spleen, have an immune response to mixed cytokines at the onset of infection. The liver is continuously exposed to a large antigenic load because of its location and function. It has an “epithelial constitution” and contains a large number of phagocytic cells, antigen-presenting cells and lymphocytes and it is a site for the production of cytokines, complement components, and acute phase proteins. In the liver, cytokines such as IFN-γ and TNF-α are involved in the activation of immunocompetent cells and account for the final microbicidal response (Murray and Nathan, 1999; Murray et al., 2005). The temporal variation in the specific immune response of these organs may be related to the differential control of the parasite load on the liver and spleen of the infected host (Kubes and Jenne, 2018). It is known that IL-10 and TGF-β are macrophage-deactivating cytokines that play key roles in murine and human VL (Wilson et al., 2005; Nylén and Sacks, 2007). Studies of experimental infection with *L. infantum* in dogs showed a significant increase in the expression of IL-10 and TGF-β in the liver and spleen, respectively (Rodríguez-Cortés et al., 2016). Our results showed that both IL-10 and TGF-β expression were more increased in C57BL/10 liver when compared to C3H/He mice, at 180 days, when an increase in parasite load was also observed. Rodríguez-Cortés et al. (2016) demonstrated a correlation between the presence of IFN-γ and the parasite load in the liver. The same was observed in our study, in C57BL/10 mice that showed high levels of IFN-γ expression, associated with high parasitism 180 days after infection. Our results demonstrated a significant expression of IFN-γ in susceptible C57BL/10 mice livers during all the experiment and regulatory cytokines (TGF-β and IL-10) in the late phase. Similar to what was observed in the spleen, *Leishmania* DNA was found in the liver, explaining the induction of cytokines in this organ.

The immunopathological characteristics of *L. amazonensis* infection show marked differences in relation to those produced by *L. major*, the prototype of the Th1/Th2 dichotomy (Afonso and Scott, 1993; Rossi-Bergmann et al., 1993). An organ-compartmentalized cytokine response against *L. amazonensis* antigens was observed in our model, in the same way as described by Lamb et al. (2005) in *L. major*. A direct association between cytokine expressions in different organs was not observed. However, we noted a mix of different cytokines produced in all organs studied, both in C57BL/10 and C3H/He mice, corroborating the observation of mixed immune responses by other authors in *L. infantum* (Rolão et al., 2007; Maia et al., 2009) and in *L. amazonensis* (Xin et al., 2007) murine models.

ECM components also play a pivotal role during *Leishmania* infection, influencing both wound healing and parasite establishment, since it presents bioactive macromolecules, which modulate cellular events such as adhesion, migration, proliferation, differentiation, and survival (Daley et al., 2008). Thus, *Leishmania* uses certain mechanisms, which can alter the organization of the matrix, favoring its entrance into macrophages and affecting the activation state of this cell (Kulkarni et al., 2008). After skin injury caused by phlebotomine bite, high levels of TGF-β are released, acting both as chemoattractants for lymphocytes, fibroblasts, monocytes, neutrophils, and other cells (Bullard et al., 2003), and as mediator of wound healing, stimulating the synthesis and contraction of ECM by fibroblasts (Bradham et al., 1991; Leask and Abraham, 2004). TGF-β1 and TGF-β2 are the most important factors in the matrix formation, stimulating collagen (especially the type I and III collagens) and proteoglycan synthesis (Szulgit et al., 2002; Köse and Waseem, 2008). Influx and activation of leukocytes and fibroblasts lead to breakdown of the normal tissue architecture. In C57BL/6 mice infected by *Leishmania*, the organization of collagen bundles is seen at the start of healing. Non-healing or chronic lesions have been related to the impairment of TGF-β signaling pathway (Kim et al., 2003; Penn et al., 2012). TGF-β knockout mice showed an impaired late-stage wound repair (e.g., collagen deposition and reepithelialization) with a higher inflammatory response and tissue necrosis when compared with wild type mice (Kulkarni and Karlsson, 1993; Bottinger et al., 1997; Abdoli et al., 2017). In our study, LN, FN, and collagens III and IV mRNA expression were enhanced in the footpad overlapping to high TGF-β expression in C57BL/10 mice, 30 days after infection. Probably, the increase of these proteins mRNA expression occurs in an attempt to recover the damaged tissue caused by parasite proliferation. In C3H/He mice, the same did not occur probably due to tissue preservation, which corroborates the histological description by our group in previous studies (Silva-Almeida et al., 2012). C57BL/10 mice also exhibited, in the late phase of infection, an enhancement of type III collagen mRNA expression in the footpad. Previous studies showed a great number of parasitized macrophages in C57BL/10 mice infected by *L. amazonensis* after 180 days (Cardoso et al., 2010). The presence of type III collagen appeared to offer support to the inflammatory cells, mainly vacuolated and parasitized macrophages as described by our group in a previous study (Abreu-Silva et al., 2004b). Our results indicate that the increase of collagen III mRNA expression is related to the presence of infected macrophages in the lesions and may be important for assessing the pathogenesis of experimental leishmaniasis.

**Figure 6:**
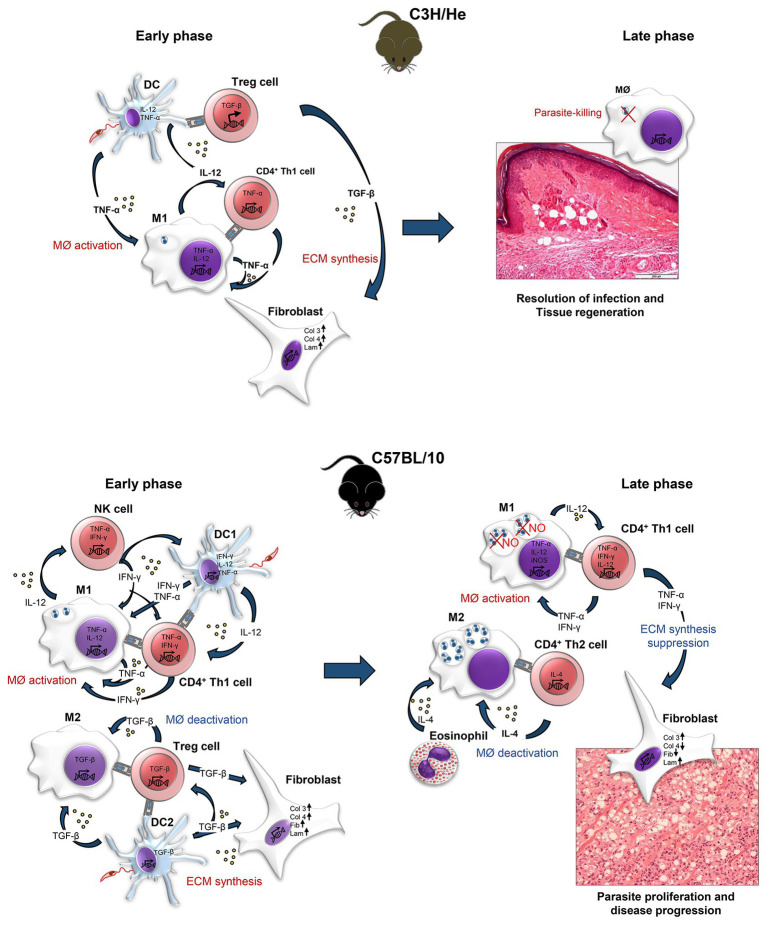
Immunopathological mechanisms occurred in the skin of C3H/He and C57BL/10 mice infected with *L. amazonensis*. In C3H/He mice, innate immune response acts quickly to eliminate *Leishmania*. Infected dendritic cells (DC1) produce TNF-α, which activates infected macrophages to kill intracellular amastigotes; and IL-12 which stimulates the differentiation of naïve CD4^+^ T cells into Th1 effectors. Th1 cells help macrophage activation with more TNF-α production. In response, macrophages produce more of these cytokines and kill intracellular amastigotes through a NO-independent mechanism. TGF-β produced by regulatory T cells acts on fibroblasts present on the infection site, stimulating the production of collagens III and IV and laminin and helping the healing process. Due to the efficient response, in the late phase of infection, no more parasites are found on the infection site and tissue structure is restored. In C57BL/10, innate immune response is also activated. IFN-γ is produced by NK cells and dendritic cells (DC1), which also produce TNF-α to active macrophages (M1) to kill intracellular amastigotes. At the same time, IL-12 produced by DC1 stimulates Th1 cells to produce more IFN-γ and TNF-α, reinforcing the parasite killing. Meanwhile, TGF-β produced by DC2 and T reg cells, stimulates macrophages alternatively activating them into the M2 profile, which are incapable of eliminating intracellular amastigotes. Upon stimulation by TGF-β, fibroblasts secrete collagen III and IV, fibronectin, and laminin in an attempt to recover the damaged tissue. In the late phase, as parasites continue to grow in the tissue, Th2 lymphocytes and eosinophils produce IL-4 stimulating M2 macrophages that are incapable of fighting infection. Amastigotes proliferate inside M2 macrophages, while M1 cells continue to fight the parasite, producing more TNF-α and IL-12. Excessive TNF-α provokes more tissue damage. Continue IFN-γ stimulus block TGF-β transcription, suppressing the fibronectin and collagen IV production by fibroblasts. The high proinflammatory stimulus attracts more macrophages to the site, which amplifies infection and destroys the tissue. DC1, dendritic cell type 1; DC2, dendritic cell type 2; ECM, extracellular matrix; MØ, macrophage; M1, classically-activated macrophage; M2, alternatively activated macrophage; NK, natural killer; T reg cells, regulatory T cells.

The findings of our study indicate that, in the primary lesion, a strong Th1 stimulus, with the upregulation of proinflammatory cytokines mRNA-transcripts in C57BL/10 infected mice when compared with C3H/He infected mice in the early and late phases of infection, suggesting an unbalance of immune response, which might contribute to excessive tissue damage in C57BL/10 strain. An upregulation of BM components (Collagen IV and FN) was also observed in the early phase of infection, mainly 1 day after *L. amazonensis* inoculation, suggesting a degradation of these components by parasite and consequent tissue invasion. In the late phase, few ECM components were expressed, either by complete wound healing, in C3H/He or by excessive tissue destruction in C57BL/10. A summary of C3H/He and C57BL/10 responses at the lesion site is illustrated in Figure 6.

In the draining lymph nodes the immune response showed a mixed pattern of cytokines expression in C57BL/10 infected mice early in infection. In the late phase of infection, there was a low cytokine expression in both mouse strains The ECM proteins expression in this organ was always low after infection in both mouse strains.

C57BL/10-infected mice liver showed high regulation of mRNA-transcripts from a mixed poll of cytokines, mainly in the late phase of infection, where parasites can be detected. In spite of liver colonization, infection caused only a mild modulation in the expression of ECM components, which suggests that tissue architecture was not intensely affected by the parasite presence.

In the spleen, *L. amazonensis* infection causes an impairment of the immune response in C57BL/10 mice during all the experiments, with higher upregulation of IL-10, mainly at later times where the parasite load is high. On the other hand, C3H/He infected mice showed an early Th1 proinflammatory response, which might have been important for infection control. Similarly to the observed in the footpad, the spleen showed an upregulation of BM components (Collagen IV and LM) in the early phase of infection, 1 day after *L. amazonensis* inoculation, mainly in C57BL/10 infected mice. C3H/He infected mice showed an upregulation of collagens expression at 30 days after infection. In the late phase of infection, despite parasite detection in this organ, there were few ECM proteins upregulated.

Overall, resistant C3H/He mice express lower cytokine levels than susceptible C57BL/10 mice, with the exception of TNF-α in the footpad and TNF-α, TGF-β, IL-12, and IFN-γ in the spleen, all in the early phase of infection. Therefore, we can presume that parasite control at the inoculation site is due to a rapid and strong innate immune response. We believe that our results will help to elucidate the role of the host immune system during *L. amazonensis* infection and wound healing by comparing two mouse strains that differ in response to infection and susceptibility to disease. The elucidation of the resistance mechanisms in the susceptible and resistant models will help us understand the natural resistance against the parasite, giving us the basis to explain the wide spectrum of diseases caused by *L. amazonensis* and contribute to the understanding of New World CL pathology in order to predict patient prognostic.

## Data Availability Statement

All datasets presented in this study are included in the article/Supplementary Material.

## Ethics Statement

The experiments with animals were conducted following the guidelines for experimental procedures of the National Council for the Control of Animal Experimentation (CONCEA) and approved by the Ethics Committee for Animal Research of the Instituto Oswaldo Cruz (CEUA/IOC), license number L001/07.

## Author Contributions

FC concepted and designed the study, as well as performed all the experiments and wrote the manuscript. TZ-V and FA-S contributed to data collection and analysis of results, assisted the writing, and contributed to the preparation of the figures. AA-S and KC contributed to funding acquisition and assisted the writing. KC administered and supervised the project. All authors contributed to manuscript revision, read, and approved the final version of the manuscript.

### Conflict of Interest

The authors declare that the research was conducted in the absence of any commercial or financial relationships that could be construed as a potential conflict of interest.
