# Supplementary material for: Modulation of Cytokines and Extracellular Matrix Proteins Expression by Leishmania amazonensis in Susceptible and Resistant Mice
Source: Front Microbiol. 2020 Aug 31;11:1986. doi: 10.3389/fmicb.2020.01986 (PMC7487551; doi:10.3389/fmicb.2020.01986)
Supplement: Supplementary file 1 [file Data_Sheet_1.docx]

**Supplementary Table 1**. Cytokines, iNOS and extracellular matrix proteins expression by quantitative RT-qPCR in the lymph nodes of C57BL/10 and C3H/He mice subcutaneously infected with 10^6^ *Leishmania amazonensis* promastigote forms in the right hind footpad.

| **Target** | **1 day** | | **30 days** | | **120 days** | | **180 days** | |
| --- | --- | --- | --- | --- | --- | --- | --- | --- |
|  | **C57BL/10** | **C3H/He** | **C57BL/10** | **C3H/He** | **C57BL/10** | **C3H/He** | **C57BL/10** | **C3H/He** |
| ***Tnf*** | 0.13 ± 0.018 | 0.08 ±0.062 | 3.78 ± 3.611^**^ | 0.15 ± 0.103 | 0.01 ± 0.001 | 0.009 ± 0.010 | 0.01 ± 0.006 | 0.001 ± 0.000 |
| ***Ifn*** | 2.24 ± 1.118 | 1.50 ± 0.071 | 41.45 ± 25.65^****^ | 1.77 ± 1.253 | 0.04 ± 0.011 | 0.008 ± 0.002 | 0.08 ± 0.093 | 0.004 ± 0.000 |
| ***Il12a*** | 0.19 ± 0.023 | 0.17 ± 0.108 | 3.66 ± 3.273^**^ | 0.18 ± 0.147 | 0.02 ± 0.007 | 0.007 ± 0.009 | 0.01 ± 0.009 | 0.001 ± 0.000 |
| ***Il4*** | 0.12 ± 0.000 | n.d. | 88.97 ± 123.8 | n.d. | 0.85 ± 0.181 | n.d. | 0.90 ± 0.252 | n.d. |
| ***Il10*** | 0.02 ± 0.007 | n.d. | 2.28 ± 3.183 | n.d. | 0.009 ± 0.0008 | n.d. | 0.02 ± 0.030 | n.d. |
| ***Tgfb1*** | 1.80 ± 0.252^*^ | 0.16 ± 0.134 | 3.78 ± 2.310^****^ | 0.45 ± 0.366 | 0.20 ± 0.025 | 0.02 ± 0.015 | 0.25 ± 0.033 | 0.002 ± 0.000 |
| ***Nos2*** | 0.27 ± 0.232^*^ | 0.01 ± 0.232 | 0.05 ± 0.044 | 0.07 ± 0.110 | 0.04 ± 0.012 | 0.008 ± 0.010 | 0.06 ± 0.080 | 0.001 ± 0.000 |
| ***Col1a1*** | 0.86 ± 0.360 | 0.15 ± 0.161 | 2.89 ± 2.207^***^ | 0.30 ±0.361 | 0.20 ± 0.080 | 0.01 ±0.013 | 0.08 ± 0.011 | 0.03 ± 0.000 |
| ***Col3a1*** | 6.04 ± 0.073 | n.d. | 38.39 ± 7.118 | n.d. | 0.92 ± 0.018 | 0.01 ± 0.012 | 0.65 ± 0.606 | n.d |
| ***Col4a2*** | 0.68 ± 0.285 | n.d. | 41.48 ± 56.33 | n.d. | 0.30 ± 0.059 | n.d. | 0.12 ± 0.049 | n.d. |
| ***Fn1*** | 0.44 ± 0.097 | n.d. | 4.29 ± 4.348 | 0.16 ± 0.015 | 0.26 ± 0.078 | n.d. | 0.45 ± 0.360 | n.d. |
| ***Lama5*** | n.d. | n.d. | n.d. | n.d. | 0.0002 ±0.0001 | n.d. | n.d | n.d. |

Expression of TNF-α, IFN-γ, IL-12, IL-4, IL-10, TGF-β, iNOS, collagens I,III and IV, laminin and fibronectin were estimated by ΔΔCT method, using RPLP0 as a reference gene. Data represents mean ± SD of two independent experiments with 4 animals assayed in triplicate. *p<0.05, **p<0.01, ***p<0.001, ****p<0.0001 comparison between strains by Two-way Anova and Bonferroni's multiple comparison test. n.d. – no detection. RQ: relative quantification. *Ifng - interferon gamma*, *Tnf - tumor necrosis factor, transcript variant 1*, *Il12a - interleukin 12a, transcript variant 1*, *Il4 - interleukin 4, transcript variant 1*, *Il10 - interleukin 10*, *Tgfb1 - transforming growth factor, beta 1, Nos2 - nitric oxide synthase 2, inducible, transcript variant 1, Col1a1 - collagen, type I, alpha 1, Col3a1 - collagen, type III, alpha 1, Col4a2 - collagen, type IV, alpha 2, Fn1 - fibronectin 1, Lama5 - laminin, alpha 5, transcript variant 1, Rplp0 - ribosomal protein, large, P0.*
